# How Does Perceived Social Support Impact Mental Health and Creative Tendencies Among Chinese Senior High School Students?

**DOI:** 10.3390/bs14111002

**Published:** 2024-10-29

**Authors:** Dongdong Gao, Yixuan Dong, Anran Kong, Xiaoyu Li

**Affiliations:** 1 Institute of Psychology and Behavior, Henan University, Kaifeng 475004, China; gaodd@henu.edu.cn (D.G.); dong123@henu.edu.cn (Y.D.); 2 The Affiliated High School of Henan University, Henan University, Kaifeng 475004, China; konganran@henu.edu.cn; 3 School of Philosophy and Public Administration, Henan University, Kaifeng 475004, China

**Keywords:** perceived social support, mental health, creative tendencies, self-esteem, stress, senior high school students

## Abstract

The senior high school period is a critical phase for the proliferation of mental health problems, as well as a key period for cognitive development among adolescents. Due to the importance of support from the external environment for students, this study aims to explore and verify the protective role of social support in the mental health and creative tendencies of senior high school students, as well as its mechanisms and boundary conditions. Based on a survey of 1463 Chinese senior high school students, a moderated mediation model was constructed. The results showed that (1) perceived social support significantly negatively predicts mental health problems and significantly positively predicts creative tendencies; (2) self-esteem mediates the impact of perceived social support on both mental health problems and creative tendencies; (3) perceived stress not only moderates the impact of self-esteem on mental health problems and creative tendencies, but also moderates the mediating effect of self-esteem. The findings of this study shed light on the positive impact of social support and the self-esteem it nurtures during the senior high school years. These insights offer valuable recommendations for practitioners aiming to prevent mental health issues and foster creative tendencies among senior high school students.

## 1. Introduction

Senior high school students are in a transitional stage from adolescence to young adulthood, characterized by rapid physiological maturation and psychological development. This makes the senior high school phase both an exciting and anxious key period [1]. Adolescence is considered a time for individuals to explore and engage in creative behaviors, developing creativity [2]. Compared to middle school students, senior high school students experience a sharp increase in cognitive and learning abilities. The development of creativity is increasingly regarded as an educational imperative [3]. Additionally, the stresses brought by academic work, intertwined with the troubles of adolescence, pose severe challenges to the mental health of senior high school students [4]. A meta-analysis on the mental health problems of Chinese senior high school students from 2010 to 2020 showed that the detection rates of mental health problems from highest to lowest were depression (28.0%), anxiety (26.3%), sleep problems (23.0%), self-harm (22.8%), suicidal ideation (17.1%), somatization (9.8%), suicide planning (6.9%), and suicide attempts (2.9%) [1], with a worsening trend over the years. Therefore, how to reduce mental health problems among senior high school students and foster their creativity, especially in the context of China’s relatively limited educational resources and the intense competition of the college entrance examination [5,6,7], becomes an important issue in senior high school education theory and practice.

Social support refers to a social network’s provision of psychological and material resources intended to benefit an individual’s ability to cope with stress [8], which aids in enhancing personal well-being and increasing resistance to health problems [8]. The rich and diverse learning and living experiences of senior high school students are inseparable from the support of family, school, peers, and other aspects, making social support of significant importance for the learning and growth of senior high school students. Previous research has shown that social support helps in controlling mental health problems [9,10,11] and aids in fostering creativity [12,13,14] and self-esteem [15]. Meanwhile, self-esteem is considered an antecedent to mental health and is closely related to individual creativity [16,17]. This implies that social support may influence the mental health and creativity of senior high school students by enhancing their self-esteem. Moreover, given the relatively tense educational resources and the universal pressure of college entrance examinations in China [5,6,7], senior high school students face more severe academic and growth pressures compared to middle school students. Whether the perception of stress by senior high school students affects the role of perceived social support in their mental health and creativity also warrants further verification. Therefore, in order to investigate the preventive factors for mental health problems and the positive factors for creativity development among senior high school students, based on the perspectives of the main effect model and the stress-buffering model of social support [18], this study explores the mechanism of the impact of perceived social support on the creative tendencies and mental health problems among senior high school students through self-esteem. Furthermore, integrating the perspectives of the ecological systems theory and the resource conservation model [19,20], it examines the moderating role of senior high school students’ perceived stress in this process.

The present study expands the existing literature in the following aspects: Firstly, it supplements the research on self-esteem as a mechanism through which social support affects mental health problems. Previous studies have examined the mediating role of self-esteem in the impact of social support on specific single mental health problems (such as depression, anxiety, etc.) [21,22,23,24]; however, senior high school students often face a variety of psychological problems [1], and specific mental health problems have limited representation for the overall mental health levels of senior high school students. Therefore, this study focuses on the overall level of psychological problems as the dependent variable, exploring the mediating role of self-esteem within the context of the general mental health among senior high school students. Secondly, the study enriches the literature on the mechanism of the impact of social support on creativity. Although previous research has separately discussed the positive effects of social support and self-esteem on creativity [12,13,25], the interaction between the two factors and their combined effect on creativity has not yet been verified, and evidence from social cognition research suggests that organized beliefs about support help frame critical cognitions about the self, including an individual’s self-esteem [15,26]. Based on this, this study explores the mechanism by which perceived social support among senior high school students enhances self-esteem, thereby affecting creative tendencies. Furthermore, considering the common pressure of college entrance examinations and academic stress among Chinese senior high school students, this study takes perceived stress as a moderating variable to further explore the boundary conditions under which perceived social support operates through self-esteem in the context of Chinese education.

## 2. Hypotheses Development

### 2.1. Perceived Social Support, Mental Health Problems, and Creative Tendencies

In nature, social support can be categorized into two types: received social support and perceived social support. Received social support refers to the specific supportive actions provided by a support network when an individual faces stress, including direct material aid and assistance from social networks and group relationships. This type of support is independent of an individual’s feelings and exists objectively. Perceived social support, on the other hand, refers to the individual’s perceived availability of social support and overall satisfaction with the support received [27]. Research indicates that the social support perceived and acknowledged by individuals has a stronger predictive effect on mental health than the objective existence of social support [28,29]. This study investigates the social support of senior high school students through perceived support.

The senior high school stage is a period with a high incidence of various mental health problems, where the troubles of adolescence intertwined with academic pressures lead to increasingly severe mental health problems among senior high school students. When they encounter negative stimuli or events, stress and stress responses are inevitably produced, followed by negative emotions such as tension, anxiety, and distress. Once the negative emotions accumulate to a certain level, mental health problems arise. The stress-buffering model of social support suggests that the belief that others will provide necessary resources may bolster one’s perceived ability to cope with demands, thus changing the appraisal of the situation and lowering its effective stress [18]. The belief that support is at hand may also dampen the unhealthy emotional and physiological responses to the event or alter maladaptive behavioral responses [30]. Meanwhile, according to the main effect model of social support, social support has a universally beneficial effect on individuals. It can promote positive psychological states (e.g., identity, purpose, self-worth, and positive affect) that induce health-promoting physiological responses.

This means that for senior high school students, support from family, school, and friends not only aids in their emotional regulation by increasing positive affect and helping to limit the intensity and duration of negative affective states [31], but it also enhances their confidence and motivation to overcome difficulties. Consequently, such support improves positive emotional experiences [32], reduces the perception of negative emotions like anxiety and depression [33], and diminishes the negative impact of adverse events on mental health. Previous research has shown that social support can enhance individual mental health while reducing the occurrence of mental health problems [9,34]. Based on this, we propose Hypothesis 1:

**H1.**  *The perceived social support among senior high school students negatively predicts mental health problems.*

Creativity is divided into eminent creativity and everyday creativity. Eminent creativity refers to “the generation of a product that is judged to be novel and also to be appropriate, useful, or valuable by a suitably knowledgeable social group” [35], suggesting that creativity is possessed only by a select few outstanding individuals. Everyday creativity, on the other hand, refers to “human originality at work and leisure across the diverse activities of everyday life” [36]. Under this definition, creativity is a universal psychological capability possessed by every healthy individual. It is a continuum from low to high, with every individual possessing creativity to a different extent, rather than an all-or-none trait. This implies that creativity can be identified and nurtured in ordinary environments such as schools, homes, and workplaces [14]. Therefore, a good external environment and atmosphere provided and ensured by schools, teachers, and parents is necessary for the development of everyday creativity in senior high school students. As a pluralistic concept, creativity involves both cognition and affect, which are considered complementary [37]. Affective creativity can be seen as an individual’s creative tendencies, which refers to the positive attitudes and psychological inclinations towards creative activities or processes that an individual possesses [38,39]. This study investigates the creativity of senior high school students from the perspective of creative tendencies.

For senior high school students, the individuals with strong creative tendencies possess a keen sense of curiosity and a desire for knowledge, independence, and autonomy in thinking, and the courage to adhere to their own views and ideas, daring to break norms and imagine boldly. Due to their novel ideas and unique perspectives, they may not be accepted or liked by teachers and classmates. However, social support can not only enhance the confidence of senior high school students to propose new views and adopt new methods to solve problems, but can also reduce the concerns of being questioned by teachers or classmates for proposing new ideas, ultimately promoting the development of creative tendencies in senior high school students. Previous research has shown that good teacher–student relationships resulting from teacher support can enhance creativity [40], and peer support also plays a positive role in promoting individuals’ creative processes [12,13]. Based on this, Hypothesis 2 is proposed:

**H2.**  *The perceived social support among senior high school students positively predicts their creative tendencies.*

### 2.2. The Mediating Role of Self-Esteem

Self-esteem is an individual’s overall sense of worth or value [41], constituting an affirmation or denial attitude towards one’s self-value, and is an essential component of an individual’s self-system. Adolescent self-esteem is influenced by various factors such as family, school, and society, among which social support is one of the significant factors affecting individual self-esteem [42]. The sociometer theory of self-esteem posits that an individual’s self-esteem primarily stems from the evaluations of others [43]. If an individual perceives acceptance and support from others, their level of self-esteem will increase. Meanwhile, Cohen noted that stress arises when one appraises a situation as threatening or demanding and does not have an appropriate coping response [18]. Appraising events as stressful often results in feelings of helplessness and threats to self-esteem. Social support precisely offers a “buffer” against this negative impact. As mentioned earlier, the perception that others can and will provide necessary resources may redefine the potential for harm posed by a situation and/or bolster one’s perceived ability to cope with the imposed demands, hence preventing a particular situation from being appraised as highly stressful. Therefore, when senior high school students perceive social support, it might positively affect their self-esteem. Research by Harter et al. shows that the quality of the interpersonal relationships between children and the significant others in their living environment is significantly related to children’s levels of self-esteem [44]. The children who are accepted have higher levels of self-esteem compared to those who often face rejection. For adolescents, previous studies have also shown that social support has a significant positive predictive effect on adolescents’ levels of self-esteem [42,45].

Furthermore, self-esteem also has various impacts on an individual’s psychological and behavioral outcomes. According to prior research [46,47], there is a close relationship between self-esteem and mental health, with significant implications for mental health status. A meta-analysis also revealed that the state of the self-esteem directly impacts the mental health status; the higher the self-esteem [17], the better the level of mental health. Individuals with high self-esteem typically approach life events with positive attitudes and emotions, thereby reducing the negative impacts of adverse life events, maintaining good moods, and promoting an improvement in mental health levels. Conversely, individuals with low self-esteem generally hold negative attitudes and evaluations about themselves, often lack confidence when dealing with life events, tend to be passive and avoidant, and over time, may even exhibit learned helplessness, thereby negatively affecting their mental health levels. From this, it can be inferred that social support, as an external environmental factor, influences mental health through the mediation of self-esteem, an internal factor. Based on this, Hypothesis 3 is proposed:

**H3.**  *Self-esteem mediates the relationship between senior high school students’ perceived social support and mental health problems.*

As a comprehensive judgment of an individual’s self-worth, self-esteem has a broad impact on an individual’s cognitive, motivational, emotional, and behavioral aspects. The “broaden-and-build” theory suggests that positive emotions can expand an individual’s attention and cognitive scope, making thinking more flexible and open and helping individuals build up lasting personal, intellectual, and psychological resources [48]. This means that the positive emotional experiences brought about by high self-esteem through social support can broaden an individual’s attention and cognitive scope, accumulating rich cognitive resources, making the individual’s thinking more flexible, their imagination richer, and their openness to information stronger, and promoting the enhancement of creative tendencies. Moreover, the higher an individual’s level of self-esteem, the less likely they are to doubt their abilities and value, actively experiencing and expressing their potential selves, thus possessing better creativity [16]. For adolescents, individuals with high self-esteem have higher self-evaluations, dare to question authority, and persist in their ideas and viewpoints, thereby facilitating the shaping of good creative tendencies [49]. Conversely, individuals with low self-esteem have lower self-evaluations, are easily disturbed by external influences, dare not stick to their viewpoints, and are not conducive to the shaping of creative tendencies [50,51]. Therefore, social support can influence senior high school students’ creative tendencies by affecting their self-esteem. Based on this, we propose Hypothesis 4:

**H4.**  *Self-esteem mediates the relationship between senior high school students’ perceived social support and creative tendencies.*

### 2.3. The Moderating Role of Perceived Stress

Lazarus believes that stress is a subjective perception that arises when the demands placed on an individual by their environment exceed their coping resources [52]. Due to individual differences, the perceptions of the same stress can vary. Given the relatively tense educational resources and the pressure of college entrance examinations in China, this study considers the perceived stress of senior high school students as a moderating variable. The ecological systems theory posits that the interaction between an individual and environmental systems affects the individual’s development [19]. In this study, an individual variable (self-esteem) as a protective factor interacts with an environmental variable (perceived stress) as a risk factor, jointly affecting an individual’s mental health problems and creative tendencies. According to the “Conservation of Resources” model [20], risk factors moderate the relationship between the resource factors and outcome variables, but the risk factors do not eliminate the beneficial effects of the resource factors, meaning that the resource factors have a favorable effect on the outcome variables regardless of whether the risk is low or high. Stress, as a risk factor, could lead to maladaptation and mental health problems. When moderating the relationship between self-esteem and mental health problems, perceived stress manifests as follows: compared with low perceived stress, senior high school students will experience more mental health problems under high perceived stress. At this point, the importance of individual self-esteem levels, as a resource factor in controlling mental health problems, is highlighted, with a stronger negative predictive effect on mental health problems. That is, whether under high or low perceived stress, senior high school students’ self-esteem levels can negatively predict mental health problems. However, compared to low perceived stress, the reduction of mental health problems with the increase of self-esteem levels is faster and the downward trend is more pronounced under high perceived stress. Based on this, Hypothesis 5 is proposed:

**H5.**  *The perceived stress of senior high school students moderates the relationship between self-esteem and mental health problems. The higher the perceived stress, the stronger the negative prediction of self-esteem on mental health problems.*

The cultivation of creative tendencies requires a relaxed environment and a mechanism for the tolerance of errors. Only by encouraging the proposal of new ideas and allowing continuous attempts and failures can a conducive creative atmosphere be fostered. When individuals feel high pressure, all their energy is focused on completing current tasks and preventing mistakes, which can decrease creative tendencies. Therefore, perceived stress negatively impacts an individual’s creative tendencies. From the perspective of the broaden-and-build theory [48], high levels of self-esteem lead to the experience of positive emotions, which in turn expand an individual’s attention, cognition, and behavioral range. This expansion helps in building the psychological resources that enhance creative tendencies. However, a state of high perceived stress is often accompanied by negative emotions [53], which cause a narrowing of cognition and attention [48]. Thus, high perceived stress impedes the positive effects of self-esteem on an individual’s attention, openness, and construction of psychological resources, leading to a reduction in the positive predictive effect of self-esteem on creative tendencies. This manifests as follows: with the increase of individual self-esteem, creative tendencies increase regardless of whether the perceived stress is high or low. However, compared to high perceived stress, the positive predictive effect of self-esteem on creative tendencies is higher under low perceived stress. Based on this, we propose Hypothesis 6:

**H6.**  *The perceived stress of senior high school students moderates the relationship between self-esteem and creative tendencies. The lower the perceived stress, the stronger the relationship between self-esteem and creative tendencies.*

### 2.4. Moderated Mediation Model

In the previous discussion, based on theories and research related to social support and self-esteem, we proposed hypotheses regarding the mediating effect of self-esteem. At the same time, we discussed the moderating role of perceived stress in the impact of senior high school students’ self-esteem on mental health problems and creative tendencies. Furthermore, we infer that the mediating effect of self-esteem is moderated by perceived stress, meaning that the latter pathways of “social support—self-esteem—mental health problems” and “social support—self-esteem—creative tendencies” are moderated by perceived stress. Therefore, we propose Hypotheses 7 and 8:

**H7.**  *The perceived stress of senior high school students moderates the mediating effect of self-esteem between social support and mental health problems. The higher the perceived stress, the stronger this mediating effect.*

**H8.**  *The perceived stress of senior high school students moderates the mediating effect of self-esteem between social support and creative tendencies. The lower the perceived stress, the stronger this mediating effect.*

**Figure 1:**
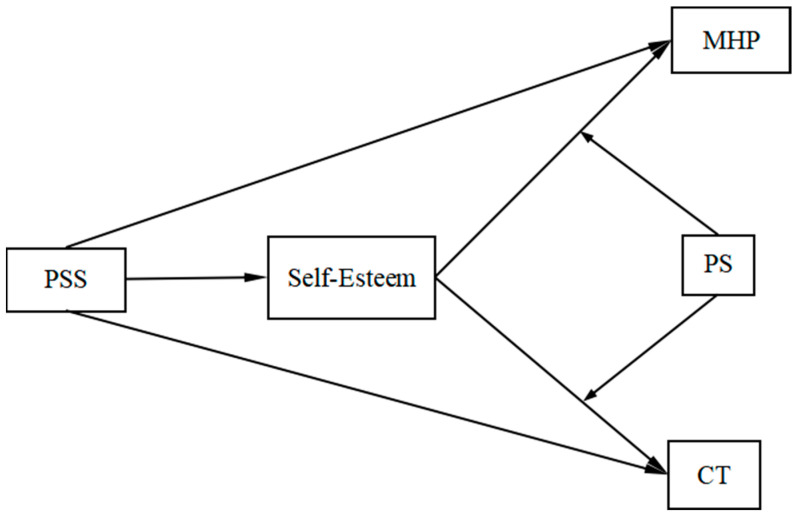
The proposed moderated mediation model; PSS, perceived social support; CT, creative tendencies; MHP, mental health problems; PS, perceived stress.

The proposed model is illustrated in Figure 1.

## 3. Materials and Methods

### 3.1. Participants

A survey was conducted among students from two senior high schools in Henan Province, covering grades 10, 11, and 12. Both schools are in urban areas, yet their student populations come from both urban and rural regions. The schools cater to both day students and boarding students. The survey was conducted by class, with homeroom teachers tasked with distributing the paper questionnaires to students in the classrooms. These teachers had received relevant training and were well acquainted with the questionnaire administration process and methodologies. The sequence of questions was identical across all questionnaires, which were collected by the homeroom teachers upon completion. The students filled out the tests voluntarily, with their express permission, anonymously and individually. In all cases, the ethical standards of research were met with an informed consent sheet. A total of 1463 questionnaires were collected. After excluding 33 invalid questionnaires (those missing answers to one question or with straight-lining responses), a total of 1430 valid questionnaires were collected. Of these, 745 were male (52.098%), and 685 were female (47.902%); there were 533 students in grade 10 (37.273%), 462 in grade 11 (32.308%), and 435 in grade 12 (30.419%); 705 students were day students (49.301%), and 725 were boarding students (50.699%); and the average age was 16.090 ± 0.986 years.

### 3.2. Measures

#### 3.2.1. Perceived Social Support Scale

The Perceived Social Support Scale (PSSS), developed by Zimet, was used to measure the participants’ perceived social support [54]. It contains 12 items, and each item is rated on a seven-point Likert scale (1 = strongly disagree, 7 = strongly agree), with higher scores indicating higher levels of social support. The sample items include “My family really tries to help me” and “I have friends with whom I can share my joys and sorrows”. In this study, the Cronbach’s alpha of this questionnaire was 0.879.

#### 3.2.2. Perceived Stress Scale

The stress dimension of the Depression Anxiety Stress Scales-21 (DASS-21), developed by Lovibond [55], was used to measure the perceived stress of the senior high school students. The scale consists of seven items, scored on a four-point scale (0 = did not apply to me at all, 3 = applied to me most of the time). The total score is the sum of all item scores, with a higher total score indicating greater perceived stress. An example item from the scale is “I found it hard to wind down”. In this study, the internal consistency coefficient of the scale was 0.837.

#### 3.2.3. Self-Esteem Scale

The self-esteem levels of the senior high school students were measured using the Self-Esteem Scale, developed by Rosenberg [56]. The scale consists of 10 items, scored on a four-point scale (1 = strongly disagree, 4 = strongly agree). After reversing the scores for the negatively worded items, the total score is the sum of all items, with higher scores indicating higher levels of self-esteem. An example item from the scale is “On the whole, I am satisfied with myself.” In this study, the scale’s internal consistency coefficient was 0.838.

#### 3.2.4. Symptom Checklist 90 (SCL-90)

The mental health problems of the senior high school students were measured by the Symptom Checklist 90 (SCL-90), developed by Derogatis [57]. The scale consists of 90 items, scored on a five-point scale (1 = not at all, 5 = extremely severe). The total score is the sum of all items, with higher scores indicating more severe psychological symptoms. An example item from the scale is “Feeling that most people cannot be trusted.” In this study, the scale’s internal consistency coefficient was 0.982.

#### 3.2.5. Adolescents’ Creative Tendencies Questionnaire

The creative tendencies of the senior high school students were measured using the Adolescents’ Creative Tendencies Questionnaire, developed by Shen [58]. The scale consists of 37 items, scored on a five-point scale (1 = strongly disagree, 5 = strongly agree). After reversing the scores for the negatively worded items, the total score is the sum of all items, with higher scores indicating stronger creative tendencies. An example item from the scale is “At school, I like to try guessing about things or problems, even if I might not guess correctly”. In this study, the scale’s internal consistency coefficient was 0.878.

#### 3.2.6. Control Variables

In our study, gender (Gender), age (Age), grade (Grade), and whether a student is a boarding student (Boarding Student) are included as control variables. For the Gender variable, 0 represents female and 1 represents male; for the Boarding Student variable, 0 indicates a day student and 1 indicates a boarding student.

### 3.3. Data Analysis

The data were first subjected to descriptive statistical analysis using SPSS 25.0, during which the Pearson correlation coefficients among the variables were calculated. Subsequently, tests for common method bias among the variables were conducted. Next, path analysis was performed using Mplus 8.3 to test the hypotheses. Finally, the effects of the moderated mediation were examined using bootstrapping with 5000 iterations to generate 95% confidence intervals. An effect was considered significant if the 95% confidence interval did not include 0 [59].

## 4. Results

### 4.1. Common Method Bias Test and Correlation Analysis

The means, standard deviations, and correlation coefficients of the variables are shown in Table 1. The correlation analysis revealed that perceived social support was significantly negatively correlated with mental health problems and perceived stress, and significantly positively correlated with creative tendencies and self-esteem. Mental health problems were significantly negatively correlated with creative tendencies and self-esteem, and significantly positively correlated with perceived stress. Creative tendencies were significantly positively correlated with self-esteem and significantly negatively correlated with perceived stress. Self-esteem was significantly negatively correlated with perceived stress. The relationships between the variables provided preliminary evidence for the hypotheses. The Harman single-factor test was used to check for common method bias [60]. The results showed that there were 24 factors with eigenvalues greater than one, and the maximum factor variance explained was 28.305%, which is less than the general empirical standard of 40%. This indicates that there is no significant common method variance in this study.

### 4.2. Hypothesis Testing

**Figure 2:**
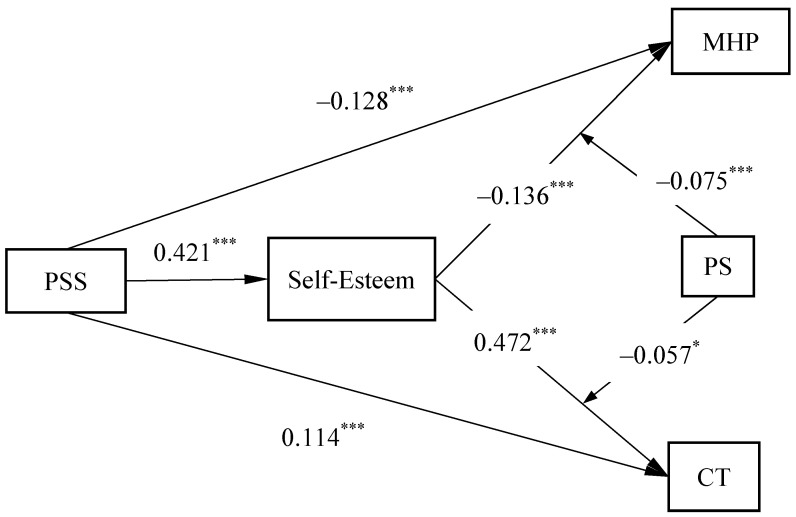
Moderated mediation model and coefficients; * *p* < 0.05, *** *p* < 0.001; PSS, perceived social support; CT, creative tendencies; MHP, mental health problems; PS, perceived stress.

Before conducting hypothesis testing, all variables were standardized. During the testing process, gender, age, grade, and boarding student were included as the control variables. The model pathways are illustrated in Figure 2.

#### 4.2.1. Direct Effect Test

To test Hypotheses 1 and 2, which involve direct effects, perceived social support was analyzed as the independent variable, with mental health problems and creative tendencies as the dependent variables. The results showed that perceived social support significantly negatively predicted mental health problems (β = −0.465, *p* < 0.001) and significantly positively predicted creative tendencies (β = 0.327, *p* < 0.001). Thus, Hypotheses 1 and 2 were supported.

#### 4.2.2. Mediating Effect Test

When perceived social support was the independent variable, mental health problems the dependent variable, and self-esteem the mediating variable, perceived social support significantly negatively predicted mental health problems (β = −0.316, *p* < 0.001) and significantly positively predicted self-esteem (β = 0.421, *p* < 0.001); self-esteem significantly negatively predicted mental health problems (β = −0.352, *p* < 0.001). The mediating effect value of self-esteem was −0.149, and *p* < 0.001, accounting for 32.043% of the total effect and supporting Hypothesis 3.

When perceived social support was the independent variable, creative tendencies the dependent variable, and self-esteem the mediating variable, perceived social support significantly positively predicted creative tendencies (β = 0.124, *p* < 0.001) and self-esteem (β = 0.421, *p* < 0.001); self-esteem significantly positively predicted creative tendencies (β = 0.483, *p* < 0.001). The mediating effect value of self-esteem was 0.204, and *p* < 0.001, accounting for 62.385% of the total effect and supporting Hypothesis 4.

#### 4.2.3. Moderating Effect Test

**Figure 3:**
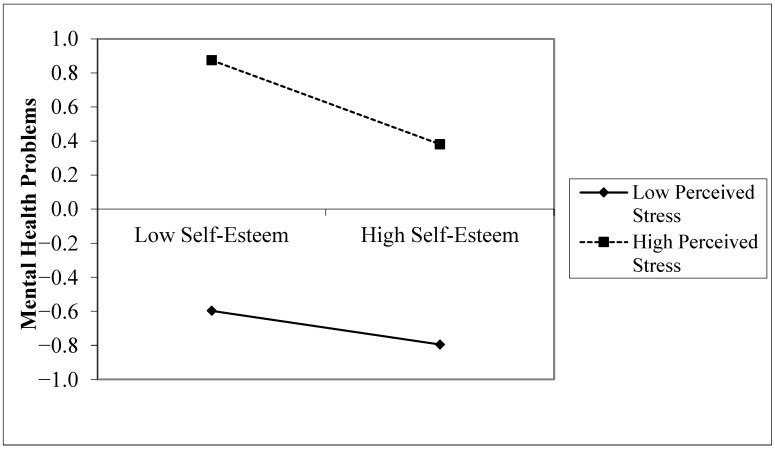
Moderating effect of perceived stress between self-esteem and mental health problems.

Testing the moderating effect of perceived stress revealed that, with self-esteem as the independent variable, mental health problems as the dependent variable, and perceived stress as the moderator, self-esteem significantly negatively predicted mental health problems (β = −0.173, *p* < 0.001), and perceived stress significantly positively predicted mental health problems (β = 0.662, *p* < 0.001). The interaction term of self-esteem and perceived stress significantly negatively predicted mental health problems (β = −0.074, *p* < 0.001). To visually demonstrate the interaction effect, simple slope analysis graphs were plotted by adding or subtracting one standard deviation from the mean of the moderator. Figure 3 shows that under low stress, as self-esteem increased, mental health problems decreased (β = −0.099, *p* < 0.001), meaning an increase of one standard deviation in self-esteem resulted in a reduction of 0.099 standard deviations in mental health problems. Under high stress, an increase in self-esteem also led to a reduction in mental health problems (β = −0.247, *p* < 0.001), but at a faster rate and more pronounced downward trend compared to low perceived stress, supporting Hypothesis 5.

**Figure 4:**
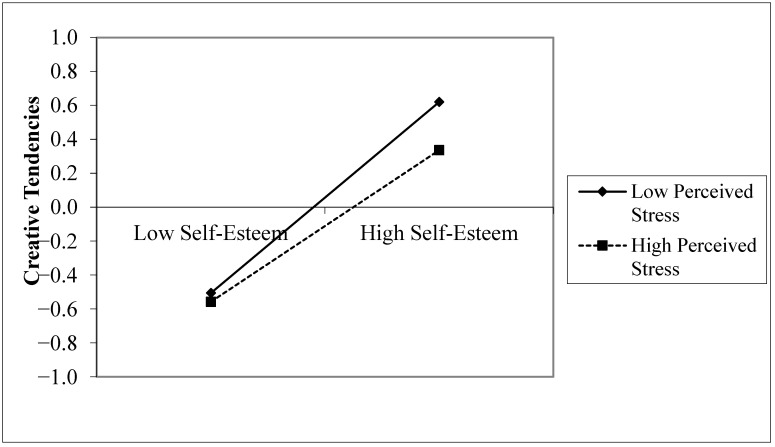
Moderating effect of perceived stress between self-esteem and creative tendencies.

With self-esteem as the independent variable and creative tendencies as the dependent variable, moderated by perceived stress, self-esteem significantly positively predicted creative tendencies (β = 0.505, *p* < 0.001), while perceived stress significantly negatively predicted creative tendencies (β = −0.084, *p* < 0.01). The interaction term of self-esteem and perceived stress significantly negatively predicted creative tendencies (β = −0.058, *p* < 0.01). Figure 4 illustrates that under low stress, as self-esteem increased, creative tendencies increased (β = 0.563, *p* < 0.001), meaning an increase of one standard deviation in self-esteem resulted in an increase of 0.563 standard deviations in creative tendencies. Under high stress, creative tendencies also increased with self-esteem (β = 0.447, *p* < 0.001), but the rate of increase was slower compared to low perceived stress, supporting Hypothesis 6.

#### 4.2.4. Test of Moderated Mediating Effects

Further testing of the final model with the moderated mediating effects was conducted, with the path coefficient results as shown in Table 2 and Figure 1. Table 3 displays the moderated mediating effect values. When the level of perceived stress was low (M − SD), the mediating effect value of self-esteem between perceived social support and psychological problems was −0.026, with a 95% bootstrap confidence interval of [−0.045, −0.006], not including 0. When the level of perceived stress was high (M + SD), the mediating effect value of self-esteem between perceived social support and psychological problems was −0.089, with a 95% bootstrap confidence interval of [−0.117, −0.062], not including 0. The difference between the two was −0.064, with a 95% confidence interval of [−0.094, −0.033], not including 0, indicating a significant difference, thus supporting Hypothesis 7. When the level of perceived stress was low (M − SD), the mediating effect value of self-esteem between perceived social support and creative tendencies was 0.223, with a 95% bootstrap confidence interval of [0.181, 0.265], not including 0. When the level of perceived stress was high (M + SD), the mediating effect value of self-esteem between perceived social support and creative tendencies was 0.175, with a 95% bootstrap confidence interval of [0.138, 0.212], not including 0. The difference between the two was −0.048, with a 95% bootstrap confidence interval of [−0.091, −0.005], not including 0, indicating a significant difference, thus supporting Hypothesis 8.

## 5. Discussion

### 5.1. The Impact of Perceived Social Support on Mental Health Problems and Creative Tendencies

This study substantiated that perceived social support could mitigate mental health problems while simultaneously fostering creative tendencies, thereby enriching the literature on the role of social support in the growth and development of senior high school students. The findings corroborate the main effect model and the stress-buffering model of social support, underscoring its positive impact on individual mental health and successful social adaptation. Previous research also demonstrated that social support plays a positive role in the mental health of senior high school students [33].

Meanwhile, for senior high school students, social support represents a crucial environmental variable. Robust social support can endow students with a heightened sense of psychological security, alleviating concerns and thus unlocking creative potential and facilitating the enhancement of creative tendencies. Previous research has indicated that environmental factors such as ample support and an open atmosphere positively influence students’ creativity [61,62]. A meta-analysis on social support and academic achievement also suggested that social support positively affects academic success [63]. The findings related to creativity in this study are consistent with the previous research.

### 5.2. The Mediating Role of Self-Esteem

Self-esteem is an individual’s positive or negative evaluation of their self-worth. In this study, social support was found to positively influence self-esteem, supporting the sociometer theory of self-esteem. According to the sociometer theory [43], self-esteem primarily derives from the extent of acceptance by others and the quality of interpersonal relationships. Social support can directly reflect an individual’s level of acceptance and the state of their interpersonal relations, thereby predicting self-esteem. If senior high school students perceive substantial social support, especially from closely related sources like family, teachers, and friends, individuals are likely to feel accepted, liked, and valued by others, leading to an increase in their level of self-esteem. Previous studies have also pointed out that social support has a positive effect on the self-esteem of adolescents [42,45].

Further, this study also confirms that social support protects the overall mental health levels of senior high school students by enhancing self-esteem. If senior high school students do not perceive social support or perceive it at a low level, they are likely to feel rejected and excluded by the group. In facing stressful situations, they are more prone to appraise these situations as threatening or demanding and lack appropriate coping responses, leading to feelings of helplessness. Consequently, their self-esteem is threatened, followed by negative emotions such as depression and anxiety. Hence, the level of social support influences individuals’ self-esteem levels and, through self-esteem, affects their mental health status. Self-esteem mediates the relationship between social support and mental health problems. Previous studies have examined the mediating role of self-esteem in the impact of social support on specific single mental health problems (depression, anxiety, etc.) [21,22,23,24], and the study by Rippon et al. also found that self-esteem and self-efficacy mediate the impact of social support on psychological well-being [64]. This study further investigates the mediating role of self-esteem within the context of general mental health problems among senior high school students.

Meanwhile, the increase in self-esteem not only enhances positive emotional experiences, such as happiness, but also fosters a proactive exploration of new things and problems, as well as perseverance in the face of difficulties and setbacks, thereby influencing the creative tendencies of senior high school students. Previous research has separately discussed the positive effects of social support and self-esteem on creativity [12,13,25]. Building on this foundation, the current study further explores the combined effects of social support and self-esteem on creativity, that is, social support indirectly affects the creativity of high school students through self-esteem. The findings of this study are consistent with previous research, where a supportive parenting style from parents influenced creativity by enhancing self-esteem [50].

### 5.3. The Moderating Role of Perceived Stress

This study discovered that the moderating role of perceived stress varies across different outcome variables. When mental health problems are the outcome variable, the relationship between self-esteem and mental health problems becomes tighter under higher perceived stress. Specifically, under lower perceived stress, as individual self-esteem increases, the mental health problems of senior high school students decrease, showing a downward trend. In contrast, under higher perceived stress, as self-esteem increases, the reduction in mental health problems accelerates, exhibiting a more pronounced downward trend. This underscores the vital role of self-esteem as a protective factor; the greater the perceived stress, the more pronounced the protective effect of self-esteem. Previous research has pointed out that stress can increase the risk of mental health problems in adolescents, while self-esteem can reduce this risk [17,65]. This study further explores the interaction between the two on the mental health of senior high school students.

Meanwhile, when creative tendencies are the outcome variable, the relationship between self-esteem and creative tendencies is tighter under lower perceived stress. To be specific, under lower perceived stress, as self-esteem increases, the creative tendencies of senior high school students increase, showing an upward trend. However, under higher perceived stress, the increase in creative tendencies is slower, indicating a more gradual upward trend. This highlights the negative impact of perceived stress as a risk factor; the greater the perceived stress, the weaker the promotive effect of self-esteem on creative tendencies. A meta-analysis based on experimental research into the relationship between stress and creativity points out that individual creative performance decreases when there is a compound source of stress (uncertainty, social evaluation) [66]. Senior high school students face complex stresses in their academic and personal lives, which include both the troubles of adolescence and the pressures related to academic advancement. The negative moderating effect of stress on the relationship between self-esteem and creativity found in this study further validates the inhibitory effect of a comprehensive perceived stress level on creativity among senior high school students.

Furthermore, in this study, social support as a situational variable, together with self-esteem as an individual variable, jointly affects individuals’ psychological and behavioral outcomes. The mediating role of self-esteem is also moderated by perceived stress. For the path “perceived social support—self-esteem—mental health problems”, the mediating role of self-esteem is more substantial under higher perceived stress. For the path “perceived social support—self-esteem—creative tendencies”, the mediating role of self-esteem is greater under lower perceived stress. The moderated mediation model constructed in this study not only explores how the protective factor of perceived social support affects mental health problems and creative tendencies, but also investigates when this effect is stronger or weaker under the influence of stress as a risk factor.

### 5.4. Practical Implications and Limitations

The research findings offer significant insights into reducing mental health problems among senior high school students and enhancing their creative tendencies.

First, it is crucial to enhance senior high school students’ perceived level of social support. Perceived social support has a universally beneficial effect on the growth and development of senior high school students, not only reducing the occurrence of mental health problems, but also facilitating the improvement of creative tendencies. Therefore, educational practices should focus on encouraging student interaction and collaboration through team projects, sports teams, and interest groups, thereby establishing a network of mutual support. Additionally, parents and teachers should provide timely verbal encouragement and feedback to students, ensuring they genuinely perceive the social support network surrounding them.

Second, it is essential to boost senior high school students’ self-esteem levels. This study demonstrates that self-esteem mediates the positive effects of social support, indicating that enhancing self-esteem can improve senior high school students’ psychological health and encourage more positive behaviors. Teachers and parents should reinforce students’ self-worth through positive feedback and recognition, affirming students’ efforts rather than just outcomes, helping them learn from setbacks, and providing timely encouragement for their progress. Also, physical education should not be overlooked, as it helps students develop sports hobbies based on their interests [67]. Additionally, students’ self-esteem also could benefit from life skills-based prevention programs designed to thwart the onset and progression of risky behaviors, while fostering essential skills such as problem-solving, effective communication, and empathy [68]. Notably, expert consultation systems based on large language models can be utilized to provide real-time, personalized support and feedback to students, parents, and teachers [69,70].

Lastly, the level of perceived stress among senior high school students should be reduced. Higher perceived stress not only increases the likelihood of mental health problems, but also hinders the enhancement of creative tendencies. Therefore, it is necessary to ensure students can identify stress and adopt effective ways to cope with it. Schools should focus on setting up workshops for relaxation and stress control techniques, such as teaching the progressive muscle relaxation technique [71], mindfulness meditation skills [72], and energy management skills [73]. Furthermore, parents and teachers should adjust the academic expectations for students, guide them to explore interests and careers, and establish academic and life goals that match their abilities and temperaments.

However, this study has the following limitations: First, the analysis was conducted using cross-sectional data, which, despite being grounded in prior theoretical and empirical research, still lacks in proving causal relationships between the variables. Future research should incorporate longitudinal study designs and behavioral experiments for a deeper exploration of the related variables. Second, due to differences in economic development levels, the scarcity of educational resources, and social and cultural backgrounds, the results of this study may not generalize well to senior high school students from other cultural contexts. Therefore, future research could consider stratified sampling among senior high school students from countries with different cultures and economic development levels to derive more comprehensive conclusions on the mechanisms and conditions of social support’s impact on students’ creative tendencies and psychological health. Further, employing meta-analysis to integrate the existing literature and explore the magnitude of the effects between variables and the potential moderating effects could offer a more comprehensive perspective on the impact of social support on mental health and creative tendencies. Finally, the instrument utilized in this study to assess mental health comprised 90 items, culminating in a survey exceeding 150 items in total. Despite the students finishing the surveys within a single senior high school class period, a potential fatigue effect cannot be discounted. Future research should contemplate employing a more concise scale that maintains the validity or randomizing the sequence of the measuring variables to mitigate the influence of the fatigue effect.

## 6. Conclusions

Perceived social support significantly negatively predicts mental health problems among senior high school students and significantly positively predicts their creative tendencies. Self-esteem mediates the impact of perceived social support on both the mental health problems and creative tendencies of senior high school students. Perceived stress moderates the effect of self-esteem on the mental health problems of senior high school students, with self-esteem having a more significant negative effect on mental health problems under high perceived stress. Perceived stress also moderates the effect of self-esteem on the creative tendencies of senior high school students, with self-esteem having a more significant positive effect on creative tendencies under low perceived stress. Perceived stress moderates the latter half of the “perceived social support—self-esteem—mental health problems” pathway, with the mediating role of self-esteem being greater under high perceived stress. Simultaneously, perceived stress moderates the latter half of the “perceived social support—self-esteem—creative tendencies” pathway, with the mediating role of self-esteem being greater under low perceived stress.

## Figures and Tables

**Table 1:** Mean, standard deviation, and correlation analysis results of each variable.

Variables	M ± SD	1	2	3	4	5
1. PSS	5.011 ± 0.964	1				
2. MHP	1.783 ± 0.618	−0.461 **	1			
3. CT	3.406 ± 0.472	0.318 **	−0.322 **	1		
4. Self-Esteem	2.961 ± 0.495	0.414 **	−0.489 **	0.545 **	1	
5. PS	1.775 ± 0.564	−0.429 **	0.763 **	−0.308 **	−0.464 **	1

** *p* < 0.01; PSS, perceived social support; CT, creative tendencies; MHP, mental health problems; PS, perceived stress.

**Table 2:** The moderated mediation model.

Predictor	Independent Variable		
	Self-Esteem	MHP	Creative Tendencies
Gender	0.066 **	0.007	0.052 *
Age	−0.024	0.009	0.011
Grade	0.128 **	0.011	0.054
Boarding Student	−0.043	0.050 **	−0.016
PSS	0.421 ***	−0.128 ***	0.114 ***
Self-Esteem		−0.136 ***	0.472 ***
Self-Esteem× PS		−0.075 ***	−0.057 *
PS		0.622 ***	−0.049
R^2^	0.192	0.627	0.320

* *p* < 0.05, ** *p* < 0.01, *** *p* < 0.001; PSS, perceived social support; CT, creative tendencies; MHP, mental health problems; PS, perceived stress.

**Table 3:** The moderated mediating effect.

Independent Variable	Value of Moderator	Indirect Effect	BootLLCI	BootULCI
MHP	Low PS (M − SD)	−0.026	−0.045	−0.006
	High PS (M + SD)	−0.089	−0.117	−0.062
	Contrast	−0.064	−0.094	−0.033
Creative Tendencies	Low PS (M − SD)	0.223	0.181	0.265
	High PS (M + SD)	0.175	0.138	0.212
	Contrast	−0.048	−0.091	−0.005

5000 bootstrapped samples; 95% bootstrapped confidence intervals; MHP, mental health problems; PS, perceived stress.

## Data Availability

The data that support the findings of this study are available from the corresponding author upon reasonable request.

## References

[1] Yu X., Zhang Y., Yu G. (2022). Prevalence of Mental Health Problems among Senior High School Students in Mainland of China from 2010 to 2020: A Meta-Analysis. Adv. Psychol. Sci..

[2] Beghetto R.A., Dilley A.E. (2016). Creative Aspirations or Pipe Dreams? Toward Understanding Creative Mortification in Children and Adolescents. New Dir. Child Adolesc. Dev..

[3] Castillo-Vergara M., Barrios Galleguillos N., Jofré Cuello L., Alvarez-Marin A., Acuña-Opazo C. (2018). Does Socioeconomic Status Influence Student Creativity?. Think. Ski. Creat..

[4] Steare T., Gutiérrez Muñoz C., Sullivan A., Lewis G. (2023). The Association between Academic Pressure and Adolescent Mental Health Problems: A Systematic Review. J. Affect. Disord..

[5] Peng H., Qi L., Wan G., Li B., Hu B. (2020). Child Population, Economic Development and Regional Inequality of Education Resources in China. Child. Youth Serv. Rev..

[6] Huang J. (2020). Operation Mechanism and Evaluation of “County High School Education Model” in the Context of Chinese College Entrance Examination System. Sci. Insights Educ. Front..

[7] Zhao D., Bai B., Bai C. (2023). Body Image Dissatisfaction and Academic Buoyancy in Adolescents: The Mediating Roles of Social Anxiety and Basic Psychological Needs Satisfaction. Psychol. Sch..

[8] Cohen S. (2004). Social Relationship and Health. Am. Psychol..

[9] Hefner J., Eisenberg D. (2009). Social Support and Mental Health Among College Students. Am. J. Orthopsychiatry.

[10] House J.S., Umberson D., Landis K.R. (1988). Structures and Processes of Social Support. Annu. Rev. Sociol..

[11] Kawachi I., Berkman L.F. (2001). Social Ties and Mental Health. J Urban Health.

[12] Choi J.N. (2012). Context and Creativity: The Theory of Planned Behavior as an Alternative Mechanism. Soc. Behav. Personal. Int. J..

[13] Liu J., You S., Jiang Y., Feng X., Song C., Yu L., Jiao L. (2023). Associations between Social Networks and Creative Behavior during Adolescence: The Mediating Effect of Psychological Capital. Think. Ski. Creat..

[14] Zhang Y., Li P., Zhang S., Xingli Z., Shi J. (2022). The Relationships of Parental Responsiveness, Teaching Responsiveness, and Creativity: The Mediating Role of Creative Self-Efficacy. Front. Psychol..

[15] Goodwin R., Costa P., Adonu J. (2004). Social Support and Its Consequences: ‘Positive’ and ‘Deficiency’ Values and Their Implications for Support and Self-esteem. Br. J. Soc. Psychol..

[16] Deng X., Zhang X. (2011). Understanding the Relationship between Self-Esteem and Creativity: A Meta-Analysis. Adv. Psychol. Sci..

[17] Gao S., Zhang X., Xu X. (2015). A Meta-Analysis of the Relationship between Self-Esteem and Mental Health: The Sample of Chinese College Students. Adv. Psychol. Sci..

[18] Cohen S., Wills T.A. (1985). Stress, Social Support, and the Buffering Hypothesis. Psychol. Bull..

[19] Bronfenbrenner U. (1994). Ecological Models of Human Development. Int. Encycl. Educ..

[20] Hobfoll S.E. (1989). Conservation of Resources: A New Attempt at Conceptualizing Stress. Am. Psychol..

[21] Bayani A.A. (2016). The Effect of Self-Esteem, Self-Efficacy and Family Social Support on Test Anxiety in Elementary Students: A Path Model. Int. J. Sch. Health.

[22] Bum C.-H., Jeon I.-K. (2016). Structural Relationships between Students’ Social Support and Self-Esteem, Depression, and Happiness. Soc. Behav. Personal. Int. J..

[23] Li M., Xu Q., Han X., Jiang Y., Ya R., Li J. (2023). A Cross-Sectional Historical Study on the Changes in Self-Esteem among Chinese Adolescents from 1996 to 2019. Front. Psychol..

[24] Shu Y., Lin W., Yang J., Huang P., Li B., Zhang X. (2022). How Social Support Predicts Anxiety among University Students during COVID-19 Control Phase: Mediating Roles of Self-esteem and Resilience. Anal. Soc. Issues Public Policy.

[25] Wang Y., Wang L. (2016). Self-Construal and Creativity: The Moderator Effect of Self-Esteem. Personal. Individ. Differ..

[26] Lakey B., Cassady P.B. (1990). Cognitive processes in perceived social support. J. Personal. Soc. Psychol..

[27] Sarason B.R., Sarason I.G., Pierce G.R. (1990). Traditional Views of Social Support and Their Impact on Assessment. Social Support: An Interactional View.

[28] Melrose K.L., Brown G.D.A., Wood A.M. (2015). When Is Received Social Support Related to Perceived Support and Well-Being? When It Is Needed. Personal. Individ. Differ..

[29] Prati G., Pietrantoni L. (2010). The Relation of Perceived and Received Social Support to Mental Health among First Responders: A Meta-Analytic Review. J. Community Psychol..

[30] Wills T.A., Cleary S.D. (1996). How Are Social Support Effects Mediated? A Test with Parental Support and Adolescent Substance Use. J. Personal. Soc. Psychol..

[31] Cohen S. (1988). Psychosocial Models of the Role of Social Support in the Etiology of Physical Disease. Health Psychol. Off. J. Div. Health Psychol. Am. Psychol. Assoc..

[32] Feeney B.C., Collins N.L. (2015). Thriving through Relationships. Curr Opin Psychol.

[33] Harandi T.F., Taghinasab M.M., Nayeri T.D. (2017). The Correlation of Social Support with Mental Health: A Meta-Analysis. Electron Physician.

[34] Qi M., Zhou S.-J., Guo Z.-C., Zhang L.-G., Min H.-J., Li X.-M., Chen J.-X. (2020). The Effect of Social Support on Mental Health in Chinese Adolescents During the Outbreak of COVID-19. J. Adolesc. Health.

[35] Sawyer R.K. (2012). Explaining Creativity: The Science of Human Innovation.

[36] Richards R. (2010). Everyday Creativity: Process and Way of Life—Four Key Issues. The Cambridge Handbook of Creativity.

[37] Williams F. (1980). Creativity Assessment Packet (CAP) Manual.

[38] Chen P.-Z., Chang T.-C., Wu C.-L. (2020). Effects of Gamified Classroom Management on the Divergent Thinking and Creative Tendency of Elementary Students. Think. Ski. Creat..

[39] Shi B., Lu Y., Dai D.Y., Lin C. (2013). Relationships between Migration to Urban Settings and Children’s Creative Inclinations. Creat. Res. J..

[40] Sun C., Zhou Z., Yu Q., Gong S., Yi L., Cao Y. (2021). Exploring the Effect of Perceived Teacher Support on Multiple Creativity Tasks: Based on the Expectancy–Value Model of Achievement Motivation. J. Creat. Behav..

[41] Rosenberg M. (1979). Conceiving the Self.

[42] Yarcheski A., Mahon N.E., Yarcheski T.J. (2001). Social Support and Well-Being in Early Adolescents: The Role of Mediating Variables. Clin. Nurs. Res..

[43] Leary M.R., Tambor E.S., Terdal S.K., Downs D.L. (1995). Self-Esteem as an Interpersonal Monitor: The Sociometer Hypothesis. J. Personal. Soc. Psychol..

[44] Harter S. (1993). Causes and Consequences of Low Self-Esteem in Children and Adolescents. Self-Esteem: The Puzzle of Low Self-Regard.

[45] Chang C.-W., Yuan R., Chen J.-K. (2018). Social Support and Depression among Chinese Adolescents: The Mediating Roles of Self-Esteem and Self-Efficacy. Child. Youth Serv. Rev..

[46] Boden J.M., Fergusson D.M., Horwood L.J. (2008). Does Adolescent Self-Esteem Predict Later Life Outcomes? A Test of the Causal Role of Self-Esteem. Dev. Psychopathol..

[47] Mann M., Hosman C., Schaalma H., Vries N. (2004). Self-Esteem in a Broad-Spectrum Approach for Mental Health Promotion. Health Educ. Res..

[48] Fredrickson B.L. (2001). The Role of Positive Emotions in Positive Psychology: The Broaden-and-Build Theory of Positive Emotions. Am. Psychol..

[49] Chen X., He J., Fan X. (2022). Relationships between Openness to Experience, Cognitive Flexibility, Self-Esteem, and Creativity among Bilingual College Students in the U.S.. Int. J. Biling. Educ. Biling..

[50] Pham H., Ng B. (2019). Self-Esteem as the Mediating Factor between Parenting Styles and Creativity. Int. J. Cogn. Behav..

[51] Alfiani I., Dwijanto D., Cahyono A.N. (2019). Mathematical Creative Thinking Ability Viewed by Self-Esteem in Problem-Based Learning with Open Ended Approach. Unnes J. Math. Educ. Res..

[52] Lazarus R.S. (1990). Theory-Based Stress Measurement. Psychol. Inq..

[53] Du J., Huang J., An Y., Xu W. (2018). The Relationship between Stress and Negative Emotion: The Mediating Role of Rumination. Clin. Res. Trials.

[54] Zimet G.D., Dahlem N.W., Zimet S.G., Farley G.K. (1988). The Multidimensional Scale of Perceived Social Support. J. Personal. Assess..

[55] Lovibond S.H., Lovibond P.F. (1995). Manual for the Depression Anxiety Stress Scales.

[56] Rosenberg M. (1965). Society and the Adolescent Self-Image.

[57] Derogatis L.R., Lipman R.S., Rickels K., Uhlenhuth E.H., Covi L. (1974). The Hopkins Symptom Checklist (HSCL): A Self-Report Symptom Inventory. Behav. Sci..

[58] Shen J., Wang X., Shi B. (2005). A Study on the Structure and Development of Adolescents’ Creative Tendencies. Psychol. Dev. Educ..

[59] Preacher K.J., Hayes A.F. (2008). Asymptotic and Resampling Strategies for Assessing and Comparing Indirect Effects in Multiple Mediator Models. Behav. Res. Methods.

[60] Podsakoff P.M., MacKenzie S.B., Lee J.-Y., Podsakoff N.P. (2003). Common Method Biases in Behavioral Research: A Critical Review of the Literature and Recommended Remedies. J. Appl. Psychol..

[61] Kanhai A., Singh B. (2017). Some Environmental and Attitudinal Characteristics as Predictors of Mathematical Creativity. Int. J. Math. Educ. Sci. Technol..

[62] van der Zanden P., Meijer P., Beghetto R. (2020). A Review Study about Creativity in Adolescence: Where Is the Social Context?. Think. Ski. Creat..

[63] Wu J., Fu H., Zhang Y. (2023). A Meta-Analysis of the Relationship between Perceived Social Support and Student Academic Achievement: The Mediating Role of Student Engagement. Adv. Psychol. Sci..

[64] Rippon D., Shepherd J., Wakefield S., Lee A., Pollet T.V. (2022). The Role of Self-Efficacy and Self-Esteem in Mediating Positive Associations between Functional Social Support and Psychological Wellbeing in People with a Mental Health Diagnosis. J. Ment. Health.

[65] Thorsén F., Antonson C., Palmér K., Berg R., Sundquist J., Sundquist K. (2022). Associations between Perceived Stress and Health Outcomes in Adolescents. Child Adolesc. Psychiatry Ment. Health.

[66] Byron K., Khazanchi S., Nazarian D. (2010). The Relationship between Stressors and Creativity: A Meta-Analysis Examining Competing Theoretical Models. J. Appl. Psychol..

[67] Gothe N.P., Erlenbach E., Engels H.-J. (2022). Exercise and Self-Esteem Model: Validity in a Sample of Healthy Female Adolescents. Curr. Psychol..

[68] Moulier V., Guinet H., Kovacevic Z., Bel-Abbass Z., Benamara Y., Zile N., Ourrad A., Arcella-Giraux P., Meunier E., Thomas F. (2019). Effects of a Life-Skills-Based Prevention Program on Self-Esteem and Risk Behaviors in Adolescents: A Pilot Study. BMC Psychol..

[69] Hafizh Z.A., Partino, Madjid A. (2023). Enhancing Student Well-Being through AI Chat GPT in the Smart Education University Learning Environment: A Preliminary Review of Research Literature. E3S Web Conf..

[70] Liu X.-Q., Zhang Z.-R. (2024). Potential Use of Large Language Models for Mitigating Students’ Problematic Social Media Use: ChatGPT as an Example. World J. Psychiatry.

[71] Allison S., Irwin Hamilton K., Yuan Y., Wallis Hague G. (2020). Assessment of Progressive Muscle Relaxation (PMR) as a Stress-Reducing Technique for First-Year Veterinary Students. J. Vet. Med. Educ..

[72] Alkoby A., Pliskin R., Halperin E., Levit-Binnun N. (2019). An Eight-Week Mindfulness-Based Stress Reduction (MBSR) Workshop Increases Regulatory Choice Flexibility. Emotion.

[73] Ghannam J., Afana A., Ho E.Y., Al-Khal A., Bylund C.L. (2020). The Impact of a Stress Management Intervention on Medical Residents’ Stress and Burnout. Int. J. Stress Manag..

